# The Impact of Perfluoroalkyl Substances on the Clinical Manifestations of Primary Sjögren Syndrome

**DOI:** 10.3390/toxics13070570

**Published:** 2025-07-05

**Authors:** Yun Zhao, Hangbiao Jin, Shetuan Hu, Songzhao Zhang, Meirong Zhao, Jing Xue

**Affiliations:** 1 Department of Rheumatology, Zhejiang University School of Medicine Second Affiliated Hospital, Hangzhou 310009, China; 2315115@zju.edu.cn; 2 Key Laboratory of Microbial Technology for Industrial Pollution Control of Zhejiang Province, College of Environment, Zhejiang University of Technology, Hangzhou 310032, China; hangbiao102@163.com (H.J.); shetuanhu001@163.com (S.H.); zhaomr@zjut.edu.cn (M.Z.); 3 Department of Laboratory Medicine, Zhejiang University School of Medicine Second Affiliated Hospital, Hangzhou 310009, China; 2196005@zju.edu.cn

**Keywords:** Sjögren syndrome, perfluoroalkyl substances, environmental factor, visceral lesions

## Abstract

Sjogren’s syndrome is an autoimmune disease that may be triggered by environmental factors. While the impact of perfluoroalkyl substances (PFASs) on the human immune system has been investigated, their specific effect on Sjogren’s syndrome remains unreported. We conducted this study to evaluate the association between PFAS exposure and clinical manifestations of pSS. In total, 136 patients with pSS and 148 healthy controls in the Second Affiliated Hospital of Zhejiang University School of Medicine were investigated. The concentrations of perfluoroundecanoic acid (PFUdA) in the pSS group were statistically significantly higher than those in the healthy control group. Compared to patients without leukopenia and thrombocytopenia, those with the condition had significantly lower concentrations of perfluorononanoic acid (PFNA). The serum levels of PFNA and perfluorodecanoic acid (PFDA) were found to be lower in patients with a high antinuclear antibody (ANA) titer compared to those with a low ANA titer. The serum levels of PFNA were found to be lower in patients who were anti-Sjögren’s syndrome A (anti-SSA)-positive compared to those who were anti-SSA-negative. These results indicate that the levels of serum PFASs may be correlated with the disease activity in pSS patients, and there might be an association between PFASs and the onset of pSS.

## 1. Introduction

**Figure 1:**
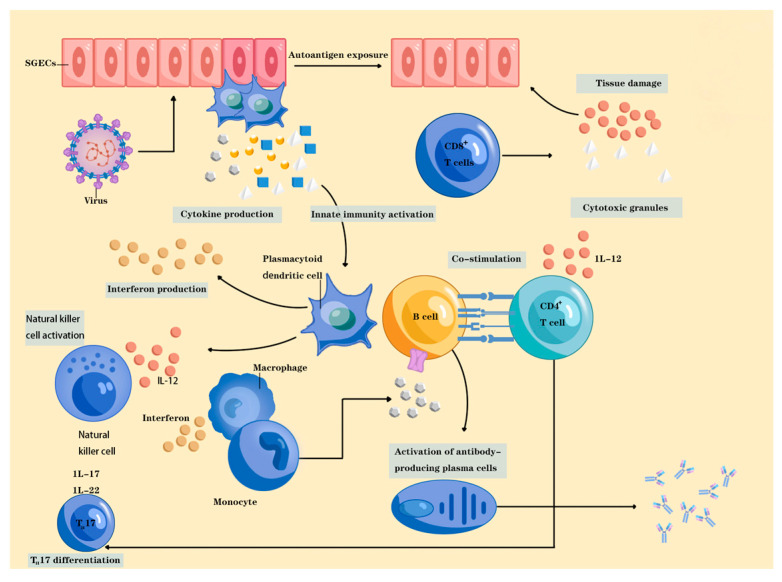
Framework diagram of the pathogenesis of Sjogren’s syndrome. Viral infection is hypothesized to induce the activation of salivary gland epithelial cells (SGECs) in genetically predisposed individuals, thereby triggering innate immune system activation and facilitating extensive crosstalk between innate and adaptive immune cells.

Sjogren syndrome (SS) is a chronic autoimmune disorder characterized by lymphocyte proliferation and the immune-mediated destruction of exocrine glands, predominantly the salivary and lacrimal glands, causing the disease hallmarks of severe dryness of the eyes and mouth, also denoted ‘sicca symptoms’ [1]. In addition to the glandular symptoms, patients with SS may also present articular, cutaneous, pulmonary, renal and muscular complications [2]. The presence of autoantibodies and hyperglobulinemia are serological characteristics observed in individuals with SS. SS is divided into secondary SS and primary SS (pSS) according to whether it is accompanied by other connective tissue diseases. The former is often secondary to systemic lupus erythematosus (SLE), rheumatoid arthritis (RA) and other pathologies. The prevalence of SS is approximately 35 million individuals worldwide, and extraglandular manifestations such as non-Hodgkin’s lymphoma develop in around 30% to 50% of patients [3]. The precise etiology and pathogenesis of pSS remain elusive. Currently, it is postulated that the action of external factors such as viral infections, sex hormones and environmental influences in individuals with genetic susceptibility, leading to immune dysregulation, is the fundamental mechanism of disease onset [4]. In genetically susceptible individuals, viral infection is thought to trigger the activation of salivary gland epithelial cells (SGECs), which results in innate immune system activation and ultimately leads to intensive crosstalk between innate and adaptive immune cells. Activated SGECs can function as antigen-presenting cells and produce a specific cytokine milieu, including IL-6, type I interferon, CXCL12, B cell activating factor and a proliferation-inducing ligand, ultimately inducing the homing of immune cells to the salivary glands, type I interferon’s overexpression by plasmacytoid dendritic cells and the promotion of B cell differentiation and survival. This causes the chronic release of autoantigens, which in turn stimulate innate immunity activation in a vicious circle [1]. Figure 1 summarizes the pathogenesis of Sjogren’s syndrome.

The health implications of chemical compounds are increasingly causing concern. Perfluoroalkyl substances (PFASs), which have been extensively manufactured and utilized across numerous commercial and industrial sectors over the past six decades, belong to a category of synthetic organic fluorine chemicals characterized by their exceptional thermal activity, chemical stability and surface reactivity [5]. The extended half-lives of PFASs, ranging from 4 to 8 years, contribute to their persistent and prolonged presence in the environment [6]. Contaminated water and food intake, indoor air inhalation and skin contact with the household environment are likely major exposure routes in human populations [7]. In the body, PFASs tend to mainly accumulate in the plasma and then in the kidney and liver [8]. The effects of PFASs in humans are predominantly linked to prenatal exposure and encompass a range of health impacts, including impaired fetal growth, low birth weight followed by postnatal catch-up growth, histopathological changes characterized by notable oxidative stress and cell apoptosis, reproductive toxicity manifested through altered hormone levels, disrupted neurodevelopment and metabolomic responses, cardiometabolic disorders, abnormal immune system function and inflammatory activities [9,10,11,12,13]. PFASs have also been associated with immune function alterations in humans. The immunomodulatory potency of PFASs has been investigated in cohort studies, revealing associations between PFASs and the dysregulation of predominantly adaptive immunity as well as adverse outcomes at the apical level, including immune-mediated diseases. A 2008–2011 epidemiological study revealed a significant increase in the incidence of ulcerative colitis correlated with higher serum levels of PFOA [14]. Autoimmune diseases associated with PFAS exposure have been documented in a population exposed to exceptionally high environmental concentrations [15]. Over the past two to three years, we have conducted extensive research on the correlation between PFASs and RA, revealing that serum PFASs concentration levels are not only associated with inflammatory factors related to RA [16,17], but also linked to specific clinical manifestations and disease activity [18] in patients with this condition. However, the effect of PFASs on pSS has not been studied.

Long-chain PFASs, such as perfluorononanoic acid (PFNA), perfluorodecanoic acid (PFDA), perfluoroundecanoic acid (PFUdA) and perfluorohexanesulfonic acid (PFHxS), containing a minimum of six carbon atoms, have been shown to be more bioaccumulative than their short-chain analogs [19] and pose more substantial health risks to both humans and other organisms [20,21]. In this study, a longitudinal clinical cohort of 136 patients with pSS was investigated at the Second Affiliated Hospital of Zhejiang University School of Medicine. Serum samples were collected from these participants during their clinic visits and analyzed for PFNA, PFDA, PFUdA and PFHxS. The main objective of this study was to explore the correlation between PFASs in pSS patients and the clinical manifestations of pSS.

## 2. Methods

### 2.1. Patients and Serum Collection

**Figure 2:**
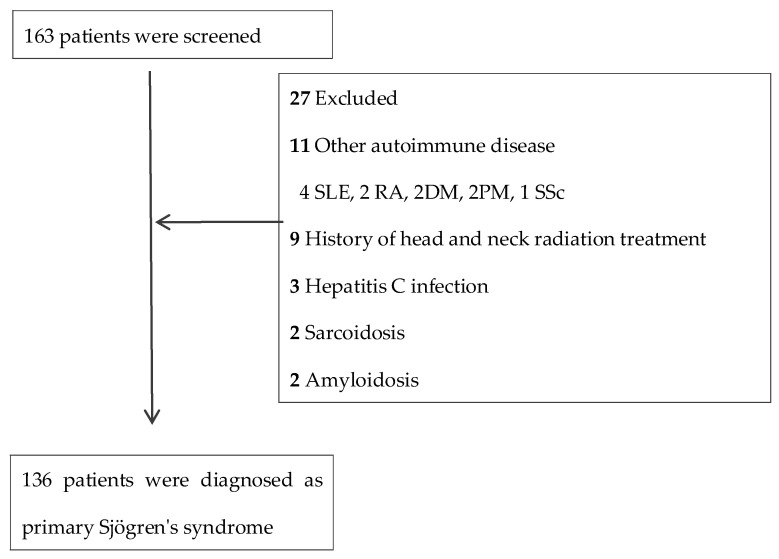
Participant flow diagram: the recruitment of patients with primary Sjögren’s syndrome. DM: dermatomyositis; PM: polymatomyositis; RA: rheumatoid arthritis; SLE: systemic lupus erythematosus; SSc: systemic sclerosis.

We designed a population-based case–control study in China and evaluated the association between PFAS exposure and the clinical manifestations of pSS. In total, 136 patients with pSS and 148 healthy controls were recruited in the Zhejiang University School of Medicine Second Affiliated Hospital between March 2021 and February 2022. Patients who had clinical symptoms of dry mouth or dry eyes and met the 2002 classification criteria for primary SS were diagnosed as pSS [22] and recruited during a routine rheumatology clinic assessment. However, patients who had other connective tissue diseases; a history of head and neck radiotherapy; or hepatitis C infection, sarcoidosis and amyloidosis were excluded. Figure 2 illustrates the inclusion and exclusion criteria and procedures. A total of 148 healthy individuals who underwent routine physical examinations at the health examination center of the hospital during the same time period were included as the healthy control group. There was no evidence that all the participants had been occupationally exposed to PFASs.

Fever is defined as a temperature greater than 37.5 °C measured by the axilla or mouth. Fatigue refers to a subjective feeling of weakness. Arthritis is defined as swelling or tenderness of the joints. Heart involvement is indicated by persistent electrocardiographic abnormalities (excluding nodal tachycardia and bradycardia) or abnormal heart structure detected through ultrasound. Pulmonary involvement is indicated by chronic diffuse interstitial infiltrates on X-ray, altered pulmonary function test results, evidence of lung alveolitis or fibrosis in computed tomography scans, and/or persistent cough and dyspnea. Interstitial Lung Disease (ILD) is defined as that reticular opacities, honeycombing, traction bronchiectasis, or ground-glass opacities can be seen on computerized tomography (CT) of the chest. Nephropathy is defined as persistent proteinuria (>0.5 g/d), altered urinalysis (hematuria, pyuria and red blood cell casts), persistently elevated serum creatinine levels (84 µmol/L), renal tubular acidosis, interstitial nephritis or glomerulonephritis. Liver involvement is indicated by abnormal serum hepatic function test results (aminotransferase, alkaline phosphatase, gamma-glutamyltransferase and bilirubin) or evidence of altered bile ducts in imaging-based examinations (ultrasound, CT or magnetic resonance imaging). Xerostomia refers to a persistent sensation of dry mouth occurring on a daily basis for more than three months or the necessity of consuming water to aid in swallowing dry food. Xerophthalmia refers to a condition characterized by persistent dryness of the eyes lasting more than three months, accompanied by recurring sensations of foreign bodies such as sand or grit in the eyes, and the necessity of using artificial tears at least three times daily. Parotid enlargement refers to an increase in parotid gland volume accompanied by a reduction in echogenicity on ultrasound imaging, often presenting with multiple hypoechoic lesions and characteristic honeycombing within the gland. Purpura refers to the extravasation of blood beneath the skin and mucous membranes, leading to non-blanching petechiae and ecchymoses. Leukopenia is defined as a white blood cell count below 4.0 × 10^9^/L on a routine blood test. Anemia is defined as a hemoglobin level lower than 110 g/L on a routine blood test. Thrombocytopenia is defined as a platelet count below 100 × 10^9^/L on a routine blood test. Transaminitis is defined as an elevated serum level of alanine aminotransferase (ALT) exceeding 50 U/L or aspartate aminotransferase (AST) exceeding 40 U/L. Hypokalemia is defined as a serum potassium ion concentration lower than 3.5 mmol/L. Low titers of ANA were defined as a titer no higher than 1:40, whereas high titers were defined as a titer of 1:80 or greater.

Medical indicators were completed on the day the patient’s blood sample was collected at the Second Affiliated Hospital of Zhejiang University Medical College. This study was approved by the Ethics Committee of the Second Affiliated Hospital of Zhejiang University School of Medicine. Blood samples were collected from the participants by physicians or nurses in BD-Vacutainer collection tubes (Becton, Dickinson and Company, West Orange, NJ, USA) from the cubital vein with an anticoagulant. After briefly mixing them, the whole blood samples were centrifuged at 3000 r/min for 10 min to separate the serum components and transferred to new blood tubes. The separated serum samples were stored in a liquid nitrogen tank and then transported to the laboratory. Prepared field blanks (200 μL of Milli-Q water, *n* = 3) were shipped with the real serum samples during the sampling process. All the collected serum specimens were stored at −80 °C until analysis for PFASs.

### 2.2. Standards, Reagents and Nomenclatures

All the certified standards were obtained from Wellington Laboratories (Guelph, ON, Canada). PFAC-MXB consists of various PFAA standard solutions, including perfluorononanoate (PFNA), perfluoroundecanoic acid (PFUdA), perfluorohexane sulfonate (PFHxS) and perfluorodecanoate (PFDA). MPFAC-MXA is a mixture of four isotopically labeled standards [23].

### 2.3. Sample Extraction

Serum samples were extracted following an acetonitrile-based extraction method adapted from a previous study [24]. Prior to extraction, 200 μL of serum sample was spiked with internal standards (1.5 ng each), and then, it was manually shaken until fully mixed. After that, 4 mL of acetonitrile was added to the serum samples. The mixture was vortexed, sonicated (53 kHz) for 30 min and centrifuged at 4000 r/min for 10 min. The supernatant was transferred to a new 10 mL polypropylene (PP) tube (Biosharp, Beijing, China). The above extraction step was repeated with 4 mL of methanol. The eluent was evaporated to near-dryness using a gentle nitrogen stream. The residue was reconstituted in 200 μL of methanol for HPLC–tandem mass spectrometry (MS/MS) analysis.

### 2.4. Instrumental Analysis

Serum samples were analyzed for 19 PFASs using high-performance liquid chromatography with tandem mass spectrometry (HPLC-MS/MS), as described in Grandjean et al. [25]. A 10 μL volume of each sample extract was injected onto an ACQUITY UPLC BEH C18 column (1.7 μm, 2.1 mm × 50 mm; Waters, agilent, Santa Clara, CA, USA) at 40 °C. The mobile phase was constituted using 2 mM ammonium acetate (Macklin, Shanghai, China) (A) and methanol (Macklin, Shanghai, China) (B). The UPLC flow rate was 0.2 mL/min, and the elution gradient started at 80% A and 20% B; it was ramped up to 50% B by 12 min and was held at 100% B for 2 min; finally, it was returned to the initial condition. The mass spectrometer (Agilent, CA, USA) was operated in electrospray negative ionization mode. Chromatograms were recorded through multiple reaction monitoring. The parent and product ions of the target PFASs are listed in Table S1 of the Supporting Information.

### 2.5. Quality Assurance and Quality Control

In order to monitor any method contamination, procedure blanks (i.e., 200 μL of Milli-Q water, n = 3) and quality control samples were processed identically to the pretreatment of the actual human serum samples. When specific PFASs were not detected in the blank samples, the limits of detection (LODs) were defined as the addition levels corresponding to signal-to-noise ratios of three. The LOD for all the PFASs measured was 0.05 ng/mL. If the analytes were detected in the blank samples, the LODs are reported as the mean concentrations plus three times the standard deviation of the blank. Triplicate recovery experiments were performed with spiked samples, including human and fetal bovine serum (Thermo Fisher Scientific, Waltham, MA, USA) samples. The mean recoveries of the main PFASs in human serum were satisfactory, ranging from 70 to 120%, and the relative standard deviations (RSDs) of the recoveries were less than 10%.

### 2.6. Statistical Analysis

Descriptive data are presented as the means ± standard deviations for continuous variables when the data were normally distributed or as M (median) (Q1 (first quartile), Q3 (third quartile)) when the data were non-normally distributed; numbers (%) are indicated for the categorical variables. Continuous variables were analyzed using Student’s *t*-test in large samples of similar variance or using the nonparametric Mann–Whitney U test for small samples. Categorical data were compared using the χ^2^ or Fisher exact test. A 2-tailed value of *p* < 0.05 indicated statistical significance. Statistical analyses were performed with the 16.0 Stata/MP program (StataCorp LP, College Station, TX, USA).

## 3. Results

### 3.1. Demographic, Clinical and Immunologic Features of 136 Patients with Primary Sjogren’s Syndrome

The patient cohort comprised 136 individuals, including 4 (2.9%) men and 132 (97.1%) women (male/female ratio = 1:33), with a mean age at onset of 41.4 ± 12.0 years (range, 20.3–70.2 year). The mean disease duration was 6.3 ± 3.7 years (range, 1.2–19.7 yr). The majority of patients initially presented with symptoms of xerostomia or xerophthalmia (71.3%), while some may have exhibited joint pain (39.7%), hypocytosis (11.0%), parotid gland enlargement (12.5%), dental caries (8.8%) or purpura (7.4%). A minority of patients were diagnosed with pSS following hypokalemic paralysis (5.1%) and fever (4.9%). The detailed patient data are presented in Table 1. There were no statistically significant differences in the epidemiological characteristics between patients with Sjögren’s syndrome and the healthy controls (Table S2). The serum concentrations of PFUdA detected were higher in pSS patients than in healthy controls (3.3 (2.8, 4.9) ng/mL vs. 1.5 (0.9, 2.3) ng/mL; *p* = 0.0117) (Table 2).

### 3.2. Correlation Between PFASs and Specific Clinical Manifestations in Patients with Primary Sjögren Syndrome

**Figure 3:**
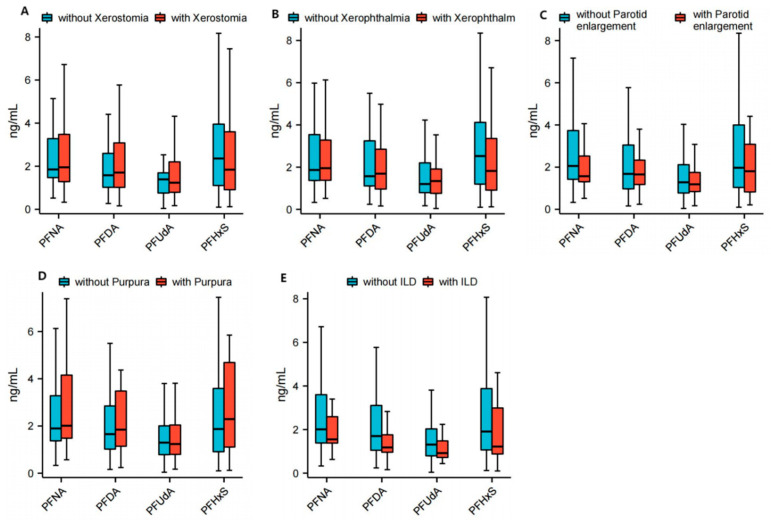
Correlation between PFASs and clinical manifestations of primary Sjögren syndrome (pSS). Correlation between PFASs, including PFNA, PFDA, PFUdA and PFHxS, and specific clinical manifestations of pSS, including xerostomia (**A**), xerophthalmia (**B**), parotid enlargement (**C**), purpura (**D**) and interstitial lung disease (ILD) (**E**) in patients with primary Sjögren syndrome. Box charts present the medians (central lines), interquartile ranges (boxes), and minimum and maximum values (whiskers). Student’s *t*-test was used. Significant *p*-value < 0.05. ILD: interstitial lung disease; PFASs: perfluoroalkyl substances; PFDA: perfluorodecanoate; PFHxS: perfluorohexane sulfonate; PFNA: perfluorononanoate; PFUdA: perfluoroundecanoate; pSS: primary Sjögren syndrome.

The levels of plasma PFNA (2.0 (1.3, 3.4) ng/mL vs. 1.9 (1.5, 3.3) ng/mL; *p* = 0.8432) and PFDA (1.7 (1.0, 3.1) ng/mL vs. 1.6 (1.0, 2.1) ng/mL; *p* = 0.4246) were found to be higher in patients with Sjogren’s syndrome presenting xerostomia compared to those without xerostomia, while the levels of PFUdA (1.2 (0.8, 2.2) ng/mL vs. 1.4 (0.7, 1.7) ng/mL; *p* = 0.7371) and PFHxS (1.8 (0.9, 3.6) ng/mL vs. 2.4 (1.1, 4.0) ng/mL; *p* = 0.5769) were lower; however, these differences did not reach statistical significance. The plasma levels of PFNA (2.0 (1.3, 3.7) ng/mL vs. 1.9 (1.4, 3.1) ng/mL; *p* = 0.8980) and PFUdA (1.2 (0.7, 2.1) ng/mL vs. 1.3 (0.8, 2.0) ng/mL; *p* = 0.7430) were higher in patients with Sjogren’s syndrome with xerophthalmia than in patients without xerophthalmia, while the level of PFHxS (1.9 (1.0, 3.9) ng/mL vs. 2.1 (0.9, 3.9) ng/mL; *p* = 0.8694) was lower, but the difference did not reach statistical significance. The plasma levels of PFNA (1.6 (1.3, 2.6) ng/mL vs. 2.1 (1.4, 3.7); *p* = 0.0979), PFUdA (1.2 (0.8, 1.8) ng/mL vs. 1.3 (0.8, 2.1); *p* = 0.6078) and PFHxS (1.8 (0.7, 3.2) ng/mL vs. 1.9 (1.0, 4.0); *p* = 0.3611) were lower in patients with Sjogren’s syndrome with parotid enlargement than in patients without parotid enlargement, but the differences were not statistically significant. The plasma levels of PFNA (2.0 (1.4, 4.3) ng/mL vs. 1.9 (1.4, 3.3); *p* = 0.7430) and PFHxS (2.3 (1.1, 4.8) ng/mL vs. 1.9 (0.9, 3.6); *p* = 0.5306) were higher in patients with Sjogren’s syndrome with purpura and lower levels of PFAud than in patients without purpura, but the difference also did not reach statistical significance. The plasma levels of PFAud (1.2 (0.9, 2.2) ng/mL vs. 1.3 (0.8, 2.0); *p* = 0.8192) were lower in patients with Sjogren’s syndrome with ILD and higher in patients without ILD, but the difference also did not reach statistical significance. The median plasma levels of PDFA were similar between pSS patients who had dry eye symptoms or parotid gland swelling or purple spots on the skin compared to pSS patients who did not have these symptoms; the median plasma levels of PNFA were similar between pSS patients who had interstitial lung disease (ILD) and those who did not have it. The detailed data are shown in Figure 3 and Table S3.

### 3.3. Correlation Between PFASs and Specific Blood Tests in Patients with Primary Sjögren Syndrome

**Figure 4:**
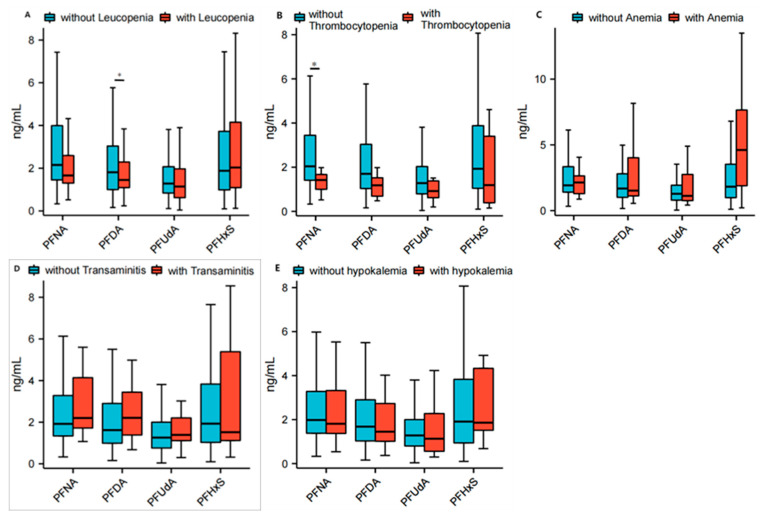
Correlation between PFASs and blood tests for primary Sjögren Syndrome (pSS). Correlation between PFASs, including PFNA, PFDA, PFUdA and PFHxS, and specific abnormal blood tests for pSS, including leukopenia (**A**), thrombocytopenia (**B**), anemia (**C**), transaminitis (**D**) and hypokalemia (**E**) in patients with primary Sjögren syndrome. Box charts present the medians (central lines), interquartile ranges (boxes), and minimum and maximum values (whiskers). Student’s *t*-test was used. * Significant *p*-value < 0.05. PFASs: perfluoroalkyl substances; PFDA: perfluorodecanoate; PFHxS: perfluorohexane sulfonate; PFNA: perfluorononanoate; PFUdA: perfluoroundecanoate; pSS: primary Sjögren syndrome.

The serum concentrations of PFNA (1.7 (1.3, 2.6) ng/mL vs. 2.2 (1.4, 4.0) ng/mL; *p* = 0.0324), PFDA (1.5 (1.1, 2.3) ng/mL vs. 1.8 (1.0, 3.1) ng/mL; *p* = 0.2698) and PFUdA (1.1 (0.6, 2.0) ng/mL vs. 1.3 (0.8, 2.1) ng/mL; *p* = 0.2792) were found to be lower in patients with Sjogren’s syndrome combined with leukopenia compared to those without leukopenia. Conversely, the concentrations of PFHxS (2.0 (1.1, 4.2) ng/mL vs. 1.9 (0.9, 3.7) ng/mL; *p* = 0.8087) were higher in patients with leukopenia than in those without. Nevertheless, except for PFNA, the other three PFASs did not reach statistical significance between the two groups. The serum concentrations of PFNA (2.1 (1.3, 2.6) ng/mL vs. 1.9 (1.4, 3.4) ng/mL; *p* = 0.8924) and PFHxS (2.0 (1.1, 4.2) ng/mL vs. 1.9 (0.9, 3.7) ng/mL; *p* = 0.8087) were found to be higher in patients with Sjogren’s syndrome combined with anemia compared to those without anemia. Conversely, the concentrations of PFDA (1.5 (1.1, 4.0) ng/mL vs. 1.7 (1.0, 2.8) ng/mL; *p* = 0.5824) and PFUdA (1.1 (0.6, 2.0) ng/mL vs. 1.3 (0.8, 2.1) ng/mL; *p* = 0.2792) were lower in patients with Sjogren’s syndrome combined with anemia than in those without. Nevertheless, the difference between the two groups did not reach statistical significance. The serum concentrations of PFNA (1.4 (1.0, 1.7) ng/mL vs. 2.0 (1.4, 3.5) ng/mL; *p* = 0.0469), PFDA (1.2 (0.7, 1.5) ng/mL vs. 1.7 (1.0, 3.1) ng/mL; *p* = 0.1210), PFUdA (0.9 (0.6, 1.4) ng/mL vs. 1.3 (0.8, 2.0); *p* = 0.2461) and PFHxS (1.2 (0.4, 3.4) ng/mL vs. 1.9 (1.0, 3.9) ng/mL; *p* = 0.3164) were found to be lower in patients with Sjogren’s syndrome combined with thrombocytopenia than in those without thrombocytopenia, but, except for PFNA, the difference between the two groups did not reach statistical significance. The serum concentrations of PFNA (2.2 (1.6, 4.1) ng/mL vs. 1.9 (1.3, 3.3) ng/mL; *p* = 0.4499), PFDA (2.2 (1.1, 4.6) ng/mL vs. 1.6 (1.0, 2.9) ng/mL; *p* = 0.2955) and PFUdA (1.4 (1.0, 2.6) ng/mL vs. 1.3 (0.8, 2.0); *p* = 0.3646) were found to be higher in patients with Sjogren’s syndrome combined with transaminitis compared to those without transaminitis. Conversely, the concentration of PFHxS (1.5 (0.9, 7.5) ng/mL vs. 1.9 (1.0, 3.8) ng/mL; *p* = 0.9169) was lower in patients with Sjogren’s syndrome combined with transaminitis than in those without. Nevertheless, the difference between the two groups did not reach statistical significance. The serum concentrations of PFNA (1.8 (1.1, 4.1) ng/mL vs. 2.0 (1.4, 3.3) ng/mL; *p* = 0.8757), PFDA (1.5 (1.0, 3.2) ng/mL vs. 1.7 (1.0, 2.9) ng/mL; *p* = 0.9329) and (1.1 (0.5, 2.8) ng/mL vs. 1.3 (0.8, 2.0); *p* = 0.5230) were found to be lower in patients with Sjogren’s syndrome combined with transaminitis than in those without transaminitis. However, the difference between the two groups did not reach statistical significance. The serum concentrations of PFNA (1.8 (1.1, 4.1) ng/mL vs. 2.0 (1.4, 3.3) ng/mL; *p* = 0.8757), PFDA (1.5 (1.0, 3.2) ng/mL vs. 1.7 (1.0, 2.9) ng/mL; *p* = 0.9329) and PFUdA (1.1 (0.5, 2.8) ng/mL vs. 1.3 (0.8, 2.0); *p* = 0.5230) were found to be lower in patients with Sjogren’s syndrome combined with hypokalemia than in those without hypokalemia. However, the difference between the two groups did not reach statistical significance. The detailed data are shown in Figure 4 and Table S4.

### 3.4. Correlation Between PFASs and Some Specific Immunologic Indexes of Primary Sjögren Syndrome

The serum concentrations of PFNA (2.9 (1.7, 4.1) ng/mL vs. 1.9 (1.3, 2.9) ng/mL; *p* = 0.0374), PFDA (2.5 (1.1, 3.6) ng/mL vs. 1.6 (1.0, 2.6) ng/mL; *p* = 0.0428), PFUdA (1.6 (0.8, 2.7) ng/mL vs. 1.3 (0.8, 1.8) ng/mL; *p* = 0.1781) and PFHxS (2.5 (1.3, 5.6) ng/mL vs. 1.8 (0.9, 3.7) ng/mL; *p* = 0.1870) were found to be higher in Sjogren’s syndrome patients with low titers of ANA compared to those with high titers of ANA. The differences in PFNA and PFDA between the two groups reached statistical significance individually. The serum concentrations of PFNA (2.9 (1.7, 4.1) ng/mL vs. 1.9 (1.3, 2.9) ng/mL; *p* = 0.0374) and PFDA (2.5 (1.1, 3.6) ng/mL vs. 1.6 (1.0, 2.6) ng/mL; *p* = 0.0428) were found to be higher in Sjogren’s syndrome patients who were anti-SSA-negative than in those who were anti-SSA-positive, and the difference in PFNA between the two groups reached statistical significance individually. Conversely, the concentration of PFHxS (1.8 (0.9, 4.1) ng/mL vs. 1.9 (1.0, 3.8) ng/mL; *p* = 0.9739) was lower in Sjogren’s syndrome patients who were anti-SSA-negative than in those who were anti-SSA-positive, but the difference did not reach statistical significance. The serum concentrations of PFNA (2.2 (1.3, 3.7) ng/mL vs. 1.8 (1.4, 2.8) ng/mL; *p* = 0.2863), PFDA (2.0 (1.0, 3.2) ng/mL vs. 1.5 (1.0, 2.3) ng/mL; *p* = 0.1511), PFUdA (1.3 (0.8, 2.3) ng/mL vs. 1.2 (0.8, 1.6) ng/mL; *p* = 0.1423) and PFHxS (2.1 (1.1, 3.8) ng/mL vs. 1.7 (0.9, 3.9) ng/mL; *p* = 0.3741) were found to be higher in Sjogren’s syndrome patients who were anti-SSB-negative than in those who were anti-SSB-positive, but the differences for all of them did not reach statistical significance. The serum concentrations of PFNA (2.0 (1.5, 4.0) ng/mL vs. 1.9 (1.2, 3.2) ng/mL; *p* = 0.4423) and PFHxS (2.1 (1.1, 4.1) ng/mL vs. 1.8 (0.9, 3.5) ng/mL; *p* = 0.2808) were found to be higher in Sjogren’s syndrome patients with normal serum IgG than in those with high levels of serum IgG (IgG ≥ 16 g/L), but the difference did not reach statistical significance. Conversely, the concentration of PFDA (1.6 (1.1, 3.4) ng/mL vs. 1.7 (0.9, 2.7) ng/mL; *p* = 0.2099) was lower in Sjogren’s syndrome patients with normal serum IgG (7 g/L ≤ IgG ≤ 16 g/L) than in those with high levels of serum IgG, but the difference did not reach statistical significance. The serum concentrations of PFNA (2.0 (1.4, 3.3) ng/mL vs. 1.7 (1.2, 4.0) ng/mL; *p* = 0.4173), PFDA (1.7 (1.1, 2.9) ng/mL vs. 1.6 (0.5, 3.1) ng/mL; *p* = 0.2804), PFUdA (1.3 (0.8, 2.0) ng/mL vs. 1.2 (0.3, 2.8) ng/mL; *p* = 0.5290) and PFHxS (1.9 (1.0, 3.8) ng/mL vs. 2.2 (0.8, 5.0) ng/mL; *p* = 0.6361) were found to be higher in Sjogren’s syndrome patients with normal serum CRP (CRP ≤ 8 mg/L) than in those with high serum levels of CRP (CRP > 8 mg/L), but the difference did not reach statistical significance.

**Figure 5:**
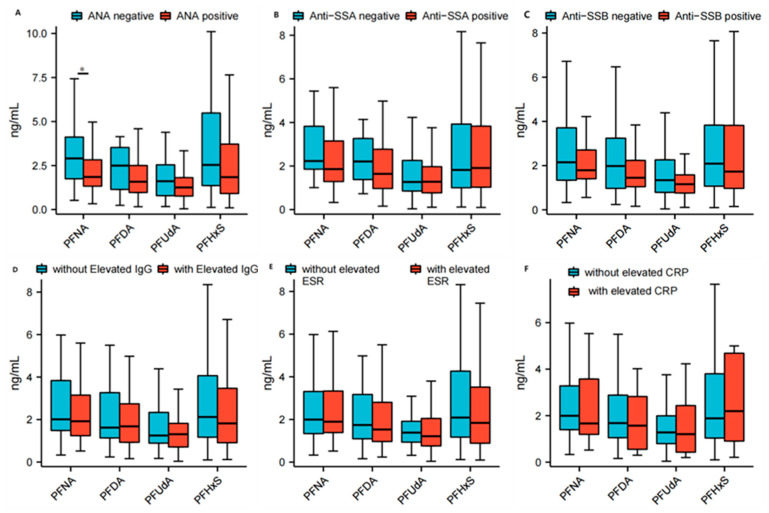
Correlation between PFASs and immunologic indexes of primary Sjögren syndrome (pSS). Correlation between PFASs, including PFNA, PFDA, PFUdA and PFHxS, and specific immunologic indexes of pSS, including antinuclear antibody (ANA) (**A**), anti-SSA (**B**), anti-SSB (**C**), immunoglobulin G (IgG) (**D**), erythrocyte sedimentation rate (ESR) (**E**) and C-reactive protein (CRP) (**F**) in patients with primary Sjögren syndrome. Box charts present the medians (central lines), interquartile ranges (boxes), and minimum and maximum values (whiskers). * Student’s *t*-test was used. Significant *p*-value < 0.05. ANA: antinuclear antibody; anti-Ro/SS-A: anti-Sjögren’s syndrome A; anti-La/SS-B: anti-Sjögren’s syndrome B; CRP: C-reactive protein; ESR: erythrocyte sedimentation rate; IgG: immunoglobulin G; PFASs: perfluoroalkyl substances; PFDA: perfluorodecanoate; PFHxS: perfluorohexane sulfonate; PFNA: perfluorononanoate; PFUdA: perfluoroundecanoate; pSS: primary Sjögren syndrome.

The serum concentrations of PFNA (2.0 (1.3, 3.3) ng/mL vs. 1.9 (1.4, 3.4) ng/mL; *p* = 0.4423), PFDA (1.7 (1.1, 3.2) ng/mL vs. 1.5 (1.0, 2.8) ng/mL; *p* = 0.4229), PFUdA (1.4 (0.9, 2.0) ng/mL vs. 1.2 (0.8, 2.1) ng/mL; *p* = 0.3783) and PFHxS (2.1 (1.2, 4.4) ng/mL vs. 1.8 (0.9, 3.5) ng/mL; *p* = 0.2177) were found to be higher in Sjogren’s syndrome patients with normal serum ESR (ESR ≤ 20 mm/h) than in those with high serum levels of ESR (ESR > 20 mm/h), but the difference did not reach statistical significance. The detailed data are shown in Figure 5 and Table S1.

## 4. Discussion

The pathogenesis of pSS is postulated to ensue upon the exposure of genetically susceptible individuals to environmental risk factors, which subsequently trigger the disease by eliciting a concerted response from both the innate and adaptive immune systems [26]. In the past, there was a greater emphasis among researchers on investigating the genetic and epigenetic aspects of autoimmune diseases. Studies in pSS have identified associations in the HLA locus as well as in >20 non-HLA loci that reach genome-wide significance (*p* <  5 × 10^−8^) [26,27]. However, the concordance rates of autoimmune diagnoses in monozygotic twins are typically below 30% [28], and the pathogenesis of autoimmune diseases may be more influenced by environmental factors rather than genetic factors [29,30]. Environmental factors, such as biological, organic and inorganic chemical factors, may facilitate the pathogenesis of SS in individuals carrying these susceptibility loci [4]. Investigations into the biological agents driving the development of SS dominate the existing literature, and infections have long been regarded as a prominent environmental risk factor. The utilization of PFASs was once prevalent in various industries and daily life, with documented implications for human health [31]. The impact of PFASs on the immune system is intricate. They can exhibit immunosuppressive effects by diminishing the production of immunoglobulins and antibodies in response to vaccines [32,33,34], while simultaneously enhancing immune responsiveness, thereby fostering the development of allergic skin disorders and asthma [35,36]. This may be attributed to the intricate structural characteristics of PFAAs, the intricacy of the immune system and the genetic background’s extensive diversity. The investigation conducted by the Genetic and Biomarkers study for Childhood Asthma (GBCA) in Taiwanese revealed that elevated serum levels of perfluoroalkyl acids (PFAAs) may induce dysregulation of Th cells and disrupt the balance of key Th1 and Th2 cytokines, ultimately contributing to asthma development with potentially greater impact on males than females. The findings of another study demonstrated a significant inverse relationship between higher concentrations of multiple PFASs and the likelihood of detecting IL-1β. Additionally, perfluoroalkyl carboxylic acids exhibited an inverse association with TNF-α, whereas perfluoroalkyl sulfonic acids showed positive associations [37]. The expression of several immunomodulatory genes (CYTL1, IL27) as well as other immune-associated genes (e.g., EMR4P, SHC4, ADORA2A) can be influenced by perfluoroalkyl substances [38].

In this study, we firstly detected the concentrations of four PFASs—PFNA, PFDA, PFUdA and PFHxS—in the serum from patients with SS and found that PFUdA was higher in the serum of SS patients than in healthy controls. The concentration of PFNA was significantly lower in patients with leukopenia, thrombocytopenia, high ANA titers and anti-SSA. Compared to our previous study [18] on the relationship between PFASs and RA in Hangzhou, the median serum concentration of PFNA was positively correlated with the disease activity. Our other study [17] showed that the mean concentrations of PFNA and PFDA in patients with RA (1.9 and 2.6 ng/mL) were higher than those in the control group (0.75 and 0.86 ng/mL). These indicated elevated exposure to PFAAs among Hangzhou residents and a further accumulation of these chemicals within the human population. Some other studies have demonstrated that PFNA exhibits immunotoxicity, which includes reduced weights of lymphoid organs, alterations in thymic and splenic lymphocyte subpopulations, atrophy of the thymus and spleen, and increased hypocellularity of the bone marrow [39,40,41]. Sjogren’s syndrome is a disease of B cell proliferation. After the excessive activation of B cells, excessive immunoglobulin and antibodies are produced. Previous studies have shown that PFASs can inhibit the production of antibodies [32,33,34], which may be the reason for the negative correlation between PFASs and some indicators of Sjogren’s syndrome in this study. Our previous study [18] showed that some PFASs were positively correlated with some indicators of RA, which is contrary to the results of the present study, which may be related to the different pathogenesis of RA. The pathophysiology of RA is characterized by the dominance of T cells, T cell-derived cytokines, and an imbalance between Th1 and Th2 responses, which plays a critical role in its pathogenesis [42,43]. Epidemiological studies have shown that PFAS exposure disrupts this balance, leading to increased Th2 and decreased Th1 cytokine production. Consequently, PFAS exposure may influence RA risk by inducing an opposing effect on the Th1/Th2 balance. However, the mechanism of PFNA immunotoxicity remains poorly understood, although one study has suggested that PFNA may exert its effects through the MAPK signaling pathway [44]. The serum levels of PFDA were found to be lower in patients with high ANA titers than in those with low ANA titers. The immunotoxicity of PFDA is similar to that of PFNA mentioned above, but it has more severe and longer-lasting toxicity in humans [45]. The immunosuppressive effect of PFDA primarily manifests as a reduction in the production of immune cells and a decrease in the mass of immune organs [45]. The mechanism of PFDA remains insufficiently investigated, although one study has proposed its potential regulatory role in NLRP3 and cIAP2 pathways [46]. Extensive research has demonstrated the potential adverse effects of PFUdA on human health, such as endocrine disruption, immunotoxicity, carcinogenicity and reproductive toxicity [32,47]. However, the present study did not reveal any significant correlation between PFUdA and the measures of SS. Oxidative stress inducing DNA damage is a possible mechanism of action for PFUnA [48]. Our present study also did not show any significant correlation between PFHxS and the measures of SS.

In an animal study, the administration of PFHxS was found to induce a reduction in the humoral immune response and alterations in immune subsets within the spleen, potentially mediated by a decrease in spleen weight, thereby indicating an immunosuppressive effect [49]. The PFHxS compound also has significant immunotoxicity and other negative health effects [49,50]. PFHxS was also found to be associated with allergy and asthma in humans [51]. In recent years, PFDA and PFHxS have also been observed to be correlated with reduced vaccine-induced antibody production [52].

Our study also has certain limitations. Firstly, it should be noted that this was a retrospective study, which restricted the reproduction of clinical data and correlation analysis between the studied PFASs and disease activity. Secondly, the pre-freezing of the serum samples may have impacted the PFAS detection. Lastly, it is important to acknowledge that this study solely serves as an observational investigation into the association between PFASs and Sjogren’s syndrome without delving into the underlying mechanisms. Therefore, further validation of these findings necessitates additional prospective studies and mechanism-oriented research.

Overall, our study demonstrated a significant association between PFASs and SS, indicating that PFASs may serve as a crucial environmental factor in promoting the initiation and progression of SS. However, it is important to acknowledge the limitations of this study, which solely focused on observing the correlation between PFASs and the clinical manifestations of SS. Furthermore, the patients included in our study may not be fully representative of the overall patient population. Consequently, additional research is warranted to validate the generalizability of our findings. And further investigations are warranted to elucidate the potential mechanisms underlying the PFASs-induced occurrence and development of SS.

## 5. Conclusions

The study found that the detected serum concentration of PFUdA was higher in SS patients than in healthy controls (3.3 (2.8, 4.9) ng/mL vs. 1.5 (0.9, 2.3) ng/mL; *p* = 0.0117). The serum concentration of PFNA (1.7 (1.3, 2.6) ng/mL vs. 2.2 (1.4, 4.0) ng/mL; *p* = 0.0324) was found to be lower in patients with Sjogren’s syndrome combined with leukopenia than in those without leukopenia. The serum concentration of PFNA (1.4 (1.0, 1.7) ng/mL vs. 2.0 (1.4, 3.5) ng/mL; *p* = 0.0469) was found to be lower in patients with Sjogren’s syndrome combined with thrombocytopenia than in those without thrombocytopenia. The serum concentration of PFNA (2.9 (1.7, 4.1) ng/mL vs. 1.9 (1.3, 2.9) ng/mL; *p* = 0.0374) was found to be higher in Sjogren’s syndrome patients with low titers of ANA than in those with high titers of ANA. The correlations between other PFASs and pSS indexes did not reach statistical significance. The mechanisms by which PFASs lead to immune disorder and, thus, the disease should be further studied.

## Figures and Tables

**Table 1:** Demographic, clinical and immunologic features of 136 Chinese patients with primary Sjögren syndrome.

Variables in Protocol *	*n* = 136
Gender, male, *n* (%)	4 (2.9)
Age at onset, mean (S.D.), years	42 ± 11
Disease duration, mean (S.D.), years	6.3 ± 3.7
Initial symptoms	
Sicca symptoms, *n* (%)	97 (71.3)
Articular involvement, *n* (%)	54 (39.7)
Parotid enlargement, *n* (%)	17 (12.5)
Hypocytosis, *n* (%)	15 (11.0)
Saprodontia, *n* (%)	12 (8.8)
Purpura, *n* (%)	10 (7.4)
Flaccid paralysis due to hypokalemia	7 (5.1)
Fever	4 (2.9)
Xerostomia, *n* (%)	104 (76.5)
Xerophthalmia, *n* (%)	84 (61.8)
Saprodontia, *n* (%)	26 (19.1)
Parotid enlargement, *n* (%)	28 (20.6)
Altered ocular tests, *n* (%)	132 (96.4)
Schirmer’s I test (≤5 mm in 5 min), *n* (%)	123 (90.4)
Ocular dye-positive, *n* (%) ^#^	114 (83.8)
Altered oral tests, *n* (%)	116 (85.3)
WUSF (≤1.5 mL in 15 min)	99 (72.8)
Parotid sialography positive ^$^	109 (94.0)
Positive salivary gland biopsy ^!^, *n* (%)	121/129 (93.8)
Arthritis	24 (17.6)
Fever	14 (10.3)
Fatigue	18 (13.2)
Purpura, *n* (%)	13 (9.6)
Flaccid paralysis due to hypokalemia	11 (8.1)
Heart involvement	44 (32.4)
Pulmonary involvement	39 (28.7)
Liver involvement	35 (25.7)
Renal involvement	13 (9.6)
Autoimmune thyroiditis	17 (12.5)
Family history of rheumatic disease	13 (9.6)
Cytopenia	69 (50.7)
Leucopenia (<4 × 10^9^/L), *n* (%)	41 (30.1)
Anemia (Hb <110 g/L), *n* (%)	17(12.5)
Thrombocytopenia (<100 × 10^9^/L), *n* (%)	17 (12.5)
Lymphopenia (<0.8 × 10^9^/L), *n* (%)	11 (8.1)
ANA positive, *n* (%)	125 (91.9)
Anti-Ro/SS-A positive, *n* (%)	106 (77.9)
Anti-La/SS-B positive, *n* (%)	65 (48.5)
RF positive, *n* (%)	89 (65.4)
High IgG levels (>17 g/L), *n* (%)	105 (77.2)
High IgA levels (>4 g/L), *n* (%)	45 (33.1)
High IgM levels (>2.3 g/L), *n* (%)	30 (22.1)
Low C3 levels (<0.9 g/L), *n* (%)	28 (20.6)
Low C4 levels (<0.1 g/L), *n* (%)	9 (6.6)

* See the definitions of clinical features in the Section 2. ^#^ Ocular dye-positive is defined as ≥4 according to van Bijsterveld’s scoring system. ^$^ Parotid sialography-positive is defined as showing the presence of diffuse sialectasias (a punctate, cavitary or destructive pattern), without evidence of obstruction in major ducts. ^!^ Positive salivary gland biopsy is defined as at least one focal lymphocyte infiltration in the biopsy labial gland tissue. ANA: antinuclear antibody; anti-Ro/SS-A: anti-Sjögren’s syndrome A; anti-La/SS-B: anti-Sjögren’s syndrome B; C3: complement 3; C4: complement 4; IgA: immunoglobulin A; IgG: immunoglobulin G; IgM: immunoglobulin M; RF: rheumatoid factors; WUSF, whole unstimulated salivary flow.

**Table 2:** Comparison of PFASs in pSS patients and healthy controls (M (Q1, Q3) ng/mL).

Group	PFNA (M (Q1, Q3))	PFDA (M (Q1, Q3))	PFUdA (M (Q1, Q3))	PFHxS (M (Q1, Q3))
Control (*n* = 148)	1.19 (0.77, 1.57)	1.9 (1.2, 3.1)	1.5 (0.9, 2.3)	2.5 (1.3, 4.3)
pSS (*n* = 136)	1.9 (1.4, 3.3)	1.7 (1.0, 3.0)	3.3 (2.8, 4.9)	1.9 (1.0, 3.9)
*p* ^&^	0.0796	0.2990	0.0117 *	0.1278

^&^ *t*-test was used; * significant = *p*-value < 0.05; M: median; Q1: first quartile; Q3: third quartile; PFASs: perfluoroalkyl substances; PFDA: perfluorodecanoate; PFHxS: perfluorohexane sulfonate; PFNA: perfluorononanoate; PFUdA: perfluoroundecanoate; pSS: primary Sjögren syndrome.

## Data Availability

The data are contained within the article. Additional data are available upon request from the corresponding authors.

## Supplementary Materials

The following supporting information can be downloaded at: https://www.mdpi.com/article/10.3390/toxics13070570/s1, Table S1: Correlation between fluorides and some specific Immunology indexes of Primary Sjögren Syndrome; Table S2: Demographic of 136 patients with primary Sjogren’s Syndrome and 148 health controls; Table S3: Correlation between fluorides and some specific clinical manifestations of Primary Sjögren Syndrome; Table S4: Correlation between fluorides and some specific abnormal blood test of Primary Sjögren Syndrome.

## Abbreviations

pSS: primary Sjögren Syndrome; RA: rheumatoid arthritis; SLE: systemic lupus erythematosus; CRP: C-reactive protein; ESR: erythrocyte sedimentation rate; HLA: human leukocyte antigen; PFASs: perfluoroalkyl substances; PFNA: perfluorononanoate; PFUdA: perfluoroundecanoic acid; PFDA: perfluorodecanoate; PFNA: perfluorononanoate; br-PFHxSK: perfluoropentanoate; PFDoA: perfluorododecanoate; PFHxS: perfluorohexane sulfonate; PFOS: perfluorooctanesulfonate
