# Supplementary material for: The Impact of Perfluoroalkyl Substances on the Clinical Manifestations of Primary Sjögren Syndrome
Source: Toxics. 2025 Jul 5;13(7):570. doi: 10.3390/toxics13070570 (PMC12300720; doi:10.3390/toxics13070570)
Supplement: Supplementary file 1 [file toxics-13-00570-s001.zip › table S1 - PSS AND FLU.pdf]

Table S1. Correlation between fluorides and some specific Immunology indexes of Primary Sjögren Syndrome

		PFNA (ng/mL)	PFDA (ng/mL)	PFUdA (ng/mL)	PFHxS (ng/mL)
Anti-nuclear antibody	≤1:40	2.9(1.7~4.1)	2.5(1.1~3.6)	1.6(0.8~2.7)	2.5(1.3~5.6)
	≥1:80	1.9(1.3~2.9)	1.6(1.0~2.6)	1.3(0.8~1.8)	1.8(0.9~3.7)
	P	0.0374	0.0428	0.1781	0.1870
Anti-SSA positive	NO	2.2(1.8~3.9)	2.2(1.3~3.4)	1.3(0.8~2.5)	1.8(0.9~4.1)
	YES	1.9(1.3~3.2)	1.6(1.0~2.8)	1.3(0.8~2.0)	1.9(1.0~3.8)
	P	0.0497	0.1521	0.5654	0.9739
Anti-SSB positive	NO	2.2(1.3~3.7)	2.0(1.0~3.2)	1.3(0.8~2.3)	2.1(1.1~3.8)
	YES	1.8(1.4~2.8)	1.5(1.0~2.3)	1.2(0.8~1.6)	1.7(0.9~3.9)
	P	0.2863	0.1511	0.1423	0.3741
Elevated IgG	NO	2.0(1.5~4.0)	1.6(1.1~3.4)	1.3(0.9~2.4)	2.1(1.1~4.1)
	YES	1.9(1.2~3.2)	1.7(0.9~2.7)	1.3(0.7~1.8)	1.8(0.9~3.5)
	P	0.4423	0.2099	0.3137	0.2808
Elevated CRP	NO	2.0(1.4~3.3)	1.7(1.1~2.9)	1.3(0.8~2.0)	1.9(1.0~3.8)
	YES	1.7(1.2~4.0)	1.6(0.5~3.1)	1.2(0.3~2.8)	2.2(0.8~5.0)
	P	0.4173	0.2804	0.5290	0.6361
Elevated ESR	NO	2.0(1.3~3.3)	1.7(1.1~3.2)	1.4(0.9~2.0)	2.1(1.2~4.4)
	YES	1.9(1.4~3.4)	1.5(1.0~2.8)	1.2(0.8~2.1)	1.8(0.9~3.5)
	P	0.4423	0.4229	0.3783	0.2177
