# Supplementary material for: The Impact of Perfluoroalkyl Substances on the Clinical Manifestations of Primary Sjögren Syndrome
Source: Toxics. 2025 Jul 5;13(7):570. doi: 10.3390/toxics13070570 (PMC12300720; doi:10.3390/toxics13070570)
Supplement: Supplementary file 1 [file toxics-13-00570-s001.zip › table S2 - PSS AND FLU.pdf]

Table S2. Demographic of 136 patients with primary Sjogren's Syndrome and 148 health controls.

Variables	pSS (n = 136)	HC (n=148)	*p*
Gender, male, n(%)	5 (3.4)	6(4.1)	0.736
Age, years (M±SD)	41±12	42±11	0.621
BMI (M±SD)	21.7±3.4	22.9±4.2	0.492

BMI: Body Mass Index; HC: health controls; M±SD: Mean ± Standard Deviation; pSS: primary Sjogren's Syndrome.
