# Supplementary material for: The Impact of Perfluoroalkyl Substances on the Clinical Manifestations of Primary Sjögren Syndrome
Source: Toxics. 2025 Jul 5;13(7):570. doi: 10.3390/toxics13070570 (PMC12300720; doi:10.3390/toxics13070570)
Supplement: Supplementary file 1 [file toxics-13-00570-s001.zip › table S3 - PSS AND FLU.pdf]

Table S3. Correlation between fluorides and some specific clinical manifestations of Primary Sjögren Syndrome

		PFNA (ng/mL)	PFDA (ng/mL)	PFUdA (ng/mL)	PFHxS (ng/mL)
	YES	2.0(1.3~3.4)	1.7(1.0~3.1)	1.2(0.8~2.2)	1.8(0.9~3.6)
Xerostomia	NO	1.9(1.5~3.3)	1.6(1.0~2.1)	1.4(0.7~1.7)	2.4(1.1~4.0)
	P	0.8432	0.4246	0.7371	0.5769
	YES	2.0(1.3~3.7)	1.7(1.0~3.0)	1.2(0.7~2.1)	1.9(1.0~3.9)
Xerophthalmia	NO	1.9(1.4~3.1)	1.7(1.1~2.9)	1.3(0.8~2.0)	2.1(0.9~3.9)
	P	0.8980	0.6989	0.7430	0.8694
	YES	1.6(1.3~2.6)	1.7(1.1~2.4)	1.2(0.8~1.8)	1.8(0.7~3.2)
Parotid enlargement	NO	2.1(1.4~3.7)	1.7(1.0~3.1)	1.3(0.8~2.1)	1.9(1.0~4.0)
	P	0.0979	0.9266	0.6078	0.3611
	YES	2.0(1.4~4.3)	1.7(1.0~2.9)	1.2(0.8~2.2)	2.3(1.1~4.8)
Purpura	NO	1.9(1.4~3.3)	1.7(1.0~3.1)	1.3(0.8~2.0)	1.9(0.9~3.6)
	P	0.7430	0.7064	0.9672	0.5306
	YES	1.9(1.7~3.5)	1.7(0.9~3.8)	1.2(0.9~2.2)	2.1(0.9~4.9)
interstitial lung disease	NO	1.9(1.3~3.3)	1.7(1.0~2.9)	1.3(0.8~2.0)	1.9(1.0~3.7)
	P	0.3047	0.5542	0.8192	0.5469
