# Supplementary material for: The Impact of Perfluoroalkyl Substances on the Clinical Manifestations of Primary Sjögren Syndrome
Source: Toxics. 2025 Jul 5;13(7):570. doi: 10.3390/toxics13070570 (PMC12300720; doi:10.3390/toxics13070570)
Supplement: Supplementary file 1 [file toxics-13-00570-s001.zip › Table S4 - PSS AND FLU.pdf]

Table S4. Correlation between fluorides and some specific abnormal blood test of Primary Sjögren Syndrome

		PFNA (ng/mL)	PFDA (ng/mL)	PFUdA (ng/mL)	PFHxS (ng/mL)
	YES	1.7(1.3~2.6)	1.5(1.1~2.3)	1.1(0.6~2.0)	2.0(1.1~4.2)
Leucopenia	NO	2.2(1.4~4.0)	1.8(1.0~3.1)	1.3(0.8~2.1)	1.9(0.9~3.7)
	P	0.0324	0.2698	0.2792	0.8087
	YES	2.1(1.3~2.6)	1.5(1.1~4.0)	1.1(0.6~2.0)	2.0(1.1~4.2)
Anemia	NO	1.9(1.4~3.4)	1.7(1.0~2.8)	1.3(0.8~2.1)	1.9(0.9~3.7)
	P	0.8924	0.5824	0.2792	0.8087
	YES	1.4(1.0~1.7)	1.2(0.7~1.5)	0.9(0.6~1.4)	1.2(0.4~3.4)
Thrombocytopenia	NO	2.0(1.4~3.5)	1.7(1.0~3.1)	1.3(0.8~2.0)	1.9(1.0~3.9)
	P	0.0469	0.1210	0.2461	0.3164
	YES	2.2(1.6~4.1)	2.2(1.1~4.6)	1.4(1.0~2.6)	1.5(0.9~7.5)
Transaminitis	NO	1.9(1.3~3.3)	1.6(1.0~2.9)	1.3(0.8~2.0)	1.9(1.0~3.8)
	P	0.4499	0.2955	0.3646	0.9169
	YES	1.8(1.1~4.1)	1.5(1.0~3.2)	1.1(0.5~2.8)	1.9(1.5~4.9)
hypokalemia	NO	2.0(1.4~3.3)	1.7(1.0~2.9)	1.3(0.8~2.0)	1.9(0.9~3.8)
	P	0.8757	0.9329	0.5230	0.4112
